# Unusually Hypervascularized Liver Lesion

**DOI:** 10.5334/jbsr.3576

**Published:** 2024-04-25

**Authors:** Eya Chaouch, Selda Aydin, Dragean Cristina Anca

**Affiliations:** 1 Cliniques universitaires Saint-Luc, BE; 2 Cliniques universitaires Saint-Luc, BE; 3 Cliniques universitaires Saint-Luc, BE

**Keywords:** Microcystic, liver, lymphangioma, MRI

## Abstract

*Teaching point:* Microcystic lymphangioma is a rare but benign lesion that should be differentiated from a neoplasm.

## Case

A 67-year-old female patient with a history of breast and lung cancer presented for a second opinion consultation for an undetermined hepatic tumor with no hepatic risk factor.

The patient first underwent a computed tomography (CT) showing a mass with heterogeneous and early arterial enhancement without wash-out. Furthermore, no signs of liver pathology (no dysmorphic liver, no portal hypertension, and the hepatic vessels were patent) were found.

**Figure 1:**
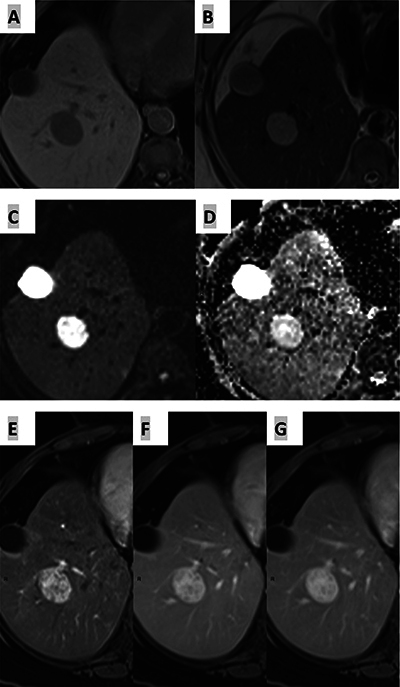
Hepatic Lesion in a 67-year-old female patient, wich is hypointense on T1 WI **(A)**, moderately hyperintense on T2 WI **(B)**, no diffusion restriction **(C)**, high ADC value **(D)** and a heterogeneous and early enhancement **(E)** on the injected sequences that was persistent in the delayed phase **(F-G)**.

A magnetic resonance imaging (MRI) scan (Figure 1) was performed and showed a round hepatic lesion (segment VI) that was moderately hyperintense on T2 weighted images and hypointense on T1 weighted images (Figure 1A and 1B). In the injected sequences, the lesion had a heterogeneous and early enhancement that was persistent in the delayed phase (Figures 1E–1G). It also revealed no diffusion restriction and a high ADC value (Figure 1C and 1D)

**Figure 2:**
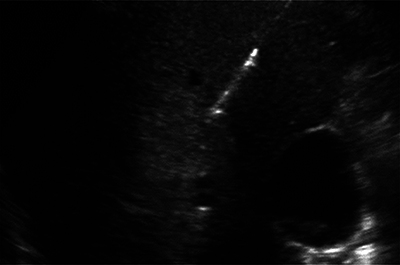
Ultrasound images of the hepatic lesion biopsy: Round hyperechoic formation with a posterior acoustic enhancement.

**Figure 3:**
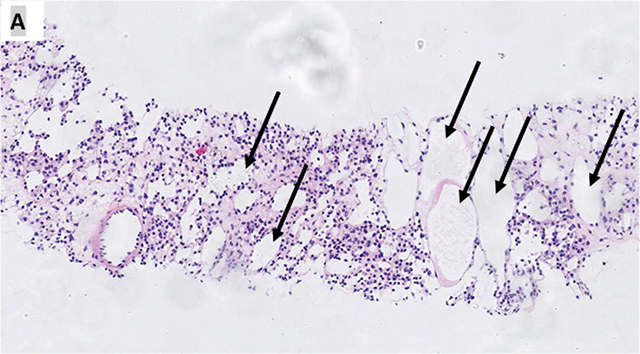

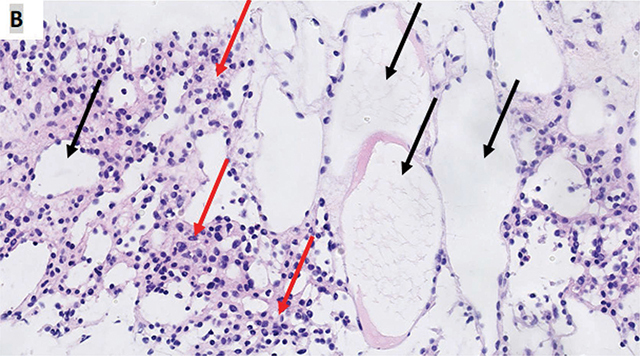

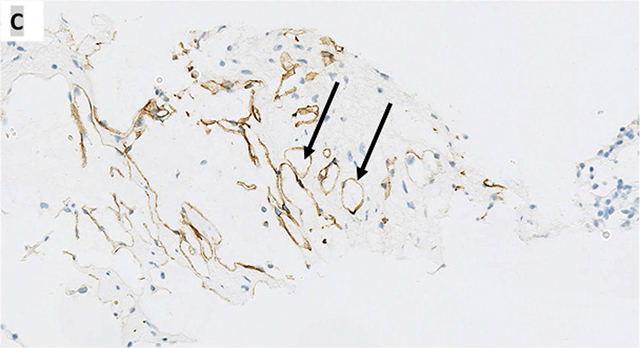
Histological slice of the biopsy showing dilated spaces lined by a single layer of non-atypical endothelial cells (black arrows) corresponding to lymphatic vessels and small hepatocytes (red arrows) **(A** and **B)**. Expression of D240 in immunohistochemistry by the lymphatic vessels **(C)**.

A biopsy on guided ultrasound was proposed (Figure 2) and showed a round hyperechoic formation with a discreet posterior acoustic enhancement. The biopsy result (Figure 3) was liver parenchyma altered by the presence of numerous dilated spaces lined by a single layer of non-atypical endothelial cells (black arrows). Small hepatocytes are located between the spaces (red arrows) (A and B, hemalun eosin staining, original magnification (A) 10× and (B) 20×). The dilated structures correspond to lymphatic vessels, which express D240 in immunohistochemistry (C, magnification 20×). The conclusion resulted that it corresponded to hepatic microcystic lymphangioma.

## Discussion

Lymphatic malformations are rare and benign congenital malformations. They consist of abnormal vessels and cystic dilatations that can have multiple superficial or deep location (retroperitoneal, splenic, hepatic . . .). They can be macrocystic, microcystic, or mixed and be present at any age, but most often occur in the pediatric population [1]. Abdominal ultrasound is often the first imaging modality for the evaluation of these patients. The microcystic form is characterized by echogenic infiltration without hypervascularization in color Doppler, sometimes with a few millimetric cysts with posterior acoustic enhancement.

MRI is the examination of choice for the evaluation of anatomical extension and for a better characterization of these lesions. A T2 sequence must be performed. It can be supplemented by a T1 sequence, allowing the identification of hemorrhagic changes, and in case of doubt, the injected sequences can be added (in our case, the injection of gadolinium enhanced the walls of the lymphatic cysts). As in ultrasound, the microcystic lymphangioma may appear as a solid lesion, and careful examination will sometimes identify small cystic components, leading to a diagnosis [1].

## References

[r1] White CL, Olivieri B, Restrepo R, McKeon B, Karakas SP, and Lee EY. Low-flow vascular malformation pitfalls: From clinical examination to practical imaging evaluation—part 1, lymphatic malformation mimickers. AJR Am J Roentgenol. 2016;206(5):940–951. DOI: 10.2214/AJR.15.15793. 26999565

